# Reversing pathologically increased EEG power by acoustic coordinated reset neuromodulation

**DOI:** 10.1002/hbm.22314

**Published:** 2013-08-01

**Authors:** Ilya Adamchic, Timea Toth, Christian Hauptmann, Peter Alexander Tass

**Affiliations:** ^1^ Institute of Neuroscience and Medicine—Neuromodulation (INM-7), Jülich Research Center, Jülich, Germany; ^2^ Department of Neuromodulation University of Cologne Cologne Germany

**Keywords:** tinnitus treatment, desynchronization, electroencephalography, non‐invasive neuromodulation, phantom perception

## Abstract

Acoustic Coordinated Reset (CR) neuromodulation is a patterned stimulation with tones adjusted to the patient's dominant tinnitus frequency, which aims at desynchronizing pathological neuronal synchronization. In a recent proof‐of‐concept study, CR therapy, delivered 4–6 h/day more than 12 weeks, induced a significant clinical improvement along with a significant long‐lasting decrease of pathological oscillatory power in the low frequency as well as *γ* band and an increase of the *α* power in a network of tinnitus‐related brain areas. As yet, it remains unclear whether CR shifts the brain activity toward physiological levels or whether it induces clinically beneficial, but nonetheless abnormal electroencephalographic (EEG) patterns, for example excessively decreased *δ* and/or *γ*. Here, we compared the patients' spontaneous EEG data at baseline as well as after 12 weeks of CR therapy with the spontaneous EEG of healthy controls by means of Brain Electrical Source Analysis source montage and standardized low‐resolution brain electromagnetic tomography techniques. The relationship between changes in EEG power and clinical scores was investigated using a partial least squares approach. In this way, we show that acoustic CR neuromodulation leads to a normalization of the oscillatory power in the tinnitus‐related network of brain areas, most prominently in temporal regions. A positive association was found between the changes in tinnitus severity and the normalization of *δ* and *γ* power in the temporal, parietal, and cingulate cortical regions. Our findings demonstrate a widespread CR‐induced normalization of EEG power, significantly associated with a reduction of tinnitus severity. *Hum Brain Mapp 35:2099–2118, 2014*. © **2013 The Authors Human Brain Mapping published by Wiley Periodicals, Inc.**

## INTRODUCTION

Subjective tinnitus is a frequent sensation of sound that cannot be attributed to an external sound source. Tinnitus may sufficiently affect everyday life, leading to sleep disorders, work disability, and psychiatric problems [Gopinath et al., 2010; Langguth et al., 2011; Schutte et al., 2009]. It is generally accepted that tinnitus generation has a central basis, typically being initiated by damage to the peripheral hearing system [De Ridder et al., 2011a,2011b; Eggermont and Roberts, 2004; Weisz et al., 2005, 2006].

In most cases, tinnitus is associated with an audiometrically measurable hearing loss that largely coincides with the tinnitus frequency range [Norena et al., 2002]. However, also in patients with a normal audiogram the presence of tinnitus may be accompanied by abnormal inner hair‐cell function [Weisz et al., 2006]. Both human and animal data show that deafferentation alters receptive fields [Dietrich et al., 2001; Irvine et al., 2001; Rauschecker, 2005] and leads to the emergence of pathological neural synchrony [Hauptmann and Tass, 2007; Merzenich et al., 1984; Ochi and Eggermont, 1997; Verkindt et al., 1995; Weisz et al., 2005] in brain regions deprived of peripheral input. Indeed, pathologically enhanced neuronal synchronization was observed in the primary auditory cortex of animals following damage to the inner ear [Hauptmann and Tass, 2007; Merzenich et al., 1984; Ochi and Eggermont, 1997] as well as in tinnitus patients [De Ridder et al., 2011b; Llinas et al., 1999; Weisz et al., 2005, 2007b].

In a magnetoencephalography (MEG) study, Weisz et al. [2005] showed a reduction of the *α* rhythm and a concomitant increase of slow‐wave (*δ*) activity, particularly in temporal regions, in a group of individuals with chronic tinnitus compared to tinnitus free controls. In fact, low‐frequency oscillations are typical for cortical regions deprived of afferent input [Steriade, 2006]. However, low frequencies are not, in general, pathological as they regularly occur during slow‐wave sleep [Benoit et al., 2000; Steriade, 2006]. In contrast, in patients suffering from chronic subjective tinnitus, one observes persistent low‐frequency activity, present in the awake state. Further studies revealed that positive symptoms may arise owing to the concerted action of slow‐ and high‐frequency oscillations [De Ridder et al., 2007; Llinas et al., 1999; Weisz et al., 2007b]. Slow‐wave activity facilitates and sustains *γ* activity that, in turn, may serve as a neural code of auditory phantom perception [De Ridder et al., 2007; Weisz et al., 2007b]. Clinical significance of this pattern of electroencephalogram (EEG) abnormality is confirmed by the fact that the tinnitus‐related distress and tinnitus loudness are correlated with this abnormal spontaneous activity pattern [De Ridder et al., 2011b; van der Loo et al., 2009; Weisz et al., 2005]. Apart from auditory cortical brain areas, nonauditory areas involved in attention and emotional regulation were also shown to be involved in the tinnitus generation, in particular, in patients with considerable amount of tinnitus distress [De Ridder et al., 2011a; Vanneste et al., 2010]. Furthermore, an altered functional connectivity between auditory and nonauditory regions seems to be a hallmark of the auditory phantom perception [De Ridder et al., 2011a; Schlee et al., 2009a]. However, these pioneering reports of altered EEG/MEG rhythmicity in tinnitus were related to a comparison between a group of tinnitus patients and a group of tinnitus free controls [Moazami‐Goudarzi et al., 2010; Weisz et al., 2005]. One limitation of these studies is that, contrary to the normal hearing control group, tinnitus patients typically have an audiometrically measurable hearing loss [Weisz et al., 2005]. Consequently, it was unclear whether the observed EEG abnormalities were specific to tinnitus, rather than being generated by the sensory deprivation owing to the hearing impairment [Weisz et al., 2005]. This point is particularly relevant as the findings by Weisz et al. (2005) are, to a certain extent, qualitatively similar to EEG findings obtained during slow‐wave sleep [Mikhailov, 1990]. Accordingly, further attempts to study the electrophysiological correlate of tinnitus in humans focused on intervention‐related changes of brain oscillations within one patient group (for review see, Langguth et al., 2012), for example, by measuring neurophysiological effects of tinnitus maskers [Kahlbrock and Weisz, 2008], auditory cortex stimulation via implanted electrodes [De Ridder et al., 2011b] or neurofeedback training [Dohrmann et al., 2007b]. In an MEG study, Kahlbrock and Weisz (2008) found a significant reduction of *δ*‐band activity in temporal areas during residual inhibition following the offset of tinnitus masker application [Kahlbrock and Weisz, 2008]. However, no tinnitus‐free controls were used in these interventional studies. It is not clear whether intervention‐induced tinnitus relief is necessarily related to a normalization of the EEG pattern. In principle, brain oscillations might be modified in such a way that tinnitus decreases, whereas EEG patterns are changed, but still remain significantly different compared to healthy controls.

In a previous study, we analyzed clinical and EEG changes caused by acoustic Coordinated Reset (CR) neuromodulation within a population of patients with chronic subjective tinnitus [Tass et al., 2012a]. As shown computationally, CR neuromodulation specifically counteracts pathological neuronal synchronization by desynchronization [Tass, 2003]. Changes of neuronal dynamics and synaptic connectivity are strongly linked in dependence on the relative timing of the pre‐ and postsynaptic spikes by the spike timing‐dependent plasticity (STDP) [Gerstner et al., 1996; Markram et al., 1997]. Already in simple neuronal networks comprising STDP, strongly synchronized states with strong synaptic connectivity may stably coexist with desynchronized states with weak synaptic connectivity [Tass and Hauptmann, 2009; Tass and Majtanik, 2006]. CR‐induced desynchronization decreases the rate of coincidences and, hence, owing to STDP also the average strength of the synaptic connections [Tass and Hauptmann, 2009; Tass and Majtanik, 2006]. Consequently, the stimulated neuronal population is shifted from a synchronized state with strong synaptic connectivity to a desynchronized state with weak connectivity [Tass and Hauptmann, 2009; Tass and Majtanik, 2006]: The network undergoes an anti‐kindling, that is, it unlearns pathological connectivity and pathological synchrony. As shown in a computational study, according to the underlying biophysics, the long‐lasting desynchronization and the unlearning of pathological connectivity (antikindling) can robustly be achieved by means of direct electrical CR stimulation or indirect, that is, synaptically mediated, excitatory, and inhibitory CR stimulation [Popovych and Tass, 2012]. Electrical CR neuromodulation caused long‐lasting desynchronization in rat hippocampal slice rendered epileptic by magnesium withdrawal [Tass et al., 2009] and sustained long‐lasting therapeutic aftereffects in MPTP monkeys [Tass et a., 2012], the standard model of experimental parkionsonism.

To specifically counteract pathological neuronal synchrony in the central auditory system, we used acoustic CR neuromodulation [Tass et al., 2012a]. To this end, based on the tonotopic organization of the central auditory system, sequences of pure tones with pitches centered around the patient's dominant tinnitus frequency are periodically delivered in an ON–OFF protocol (**METHODS**) [Tass and Popovych, 2012; Tass et al., 2012a]. Treatment with CR neuromodulation resulted in a highly significant decrease of tinnitus symptoms as measured by visual analog scale (VAS) and tinnitus questionnaire (TQ) scores [Tass et al., 2012a]. According to VAS [Adamchic et al., 2012a] and TQ [Adamchic et al., 2012b] evaluation studies, this improvement is not only statistically, but also clinically significant. In contrast, placebo treatment did not lead to any significant changes. Furthermore, after 12 weeks of acoustic CR neuromodulation *δ* and *γ* activity were significantly decreased in primary and secondary auditory cortex and in frontal areas combined with an increase of the initially reduced *α* power in auditory and prefrontal areas [Tass et al., 2012a]. However, in that study, EEG markers were compared in one patient population before and after CR therapy [Tass et al., 2012a]. Strictly speaking, it was, thus, not possible to judge whether the therapy shifted the EEG markers closer to what is supposed to be physiological (as represented by a control group of healthy subjects) or whether a completely different pattern of EEG markers evolved, for example characterized by significantly reduced *δ* and/or *γ* power as opposed to controls.

Although neural synchronization plays a fundamental role in the pathophysiology of the auditory phantom perception, up to our knowledge, as yet no study has investigated therapy‐induced changes of EEG patterns in tinnitus patients as compared to physiological reference EEG patterns recorded from a group of tinnitus‐free controls. In this study, we set out to overcome this shortcoming. To further our understanding of the pathophysiology of chronic subjective tinnitus and of the mechanisms of acoustic CR neuromodulation, the goals of this study are as follows: (i) To statistically discriminate between naïve tinnitus patients and tinnitus‐free controls on the basis of EEG spectral parameters. (ii) To compare the EEG pattern in the tinnitus patient population after 12 weeks of CR therapy with the EEG pattern of the tinnitus‐free controls. (iii) To explore relationships between CR therapy‐induced changes of different resting EEG parameters (i.e., power changes in different frequency bands observed in different brain areas) on the one hand and tinnitus symptoms on the other hand. In particular, to study whether acoustic CR neuromodulation normalizes the EEG pattern in the tinnitus patients (i.e., shifts the EEG patterns closer to a physiological pattern), or whether clinical improvement is associated with a significantly different, nonphysiological EEG pattern.

To our knowledge, this is the first study that investigates whether treatment‐induced long‐lasting changes of resting EEG spectral parameters contribute to a normalization of oscillatory brain activity and if so in which cortical brain regions normalization of EEG spectral parameters takes place. To test this, we combined a cross‐sectional with a longitudinal approach. To assess the relationship between the normalization of oscillatory activity in different brain areas and the reduction of tinnitus severity, we used the partial least‐squares (PLS) multivariate approach that holds specific advantages over conventional univariate approaches [McIntosh et al., 1996].

## MATERIALS AND METHODS

### Patients

Here, we analyze EEG data recorded in tinnitus patients who participated in a multicentric randomized, controlled clinical trial on Acoustic CR Neuromodulation in the Treatment of chronic subjective tonal tinnitus, performed in Germany between 2009 and 2010 (“RESET study,” http://ClinicalTrials.gov Identifier: NCT00927121). In total, 63 patients with chronic subjective tonal tinnitus participated in the RESET study. All patients were informed about the scope and aim of the study and a written consent was obtained from all tinnitus patients according to the declaration of Helsinki and the study was approved by the ethics commission. Patients with pulsatile, ringing, buzzing, roaring, or hissing tinnitus as well as patients with a need for hearing aids, a history of auditory hallucinations, Méniere's disease, diagnosed neurological or mental disorders, and patients taking CNS‐acting medication or participating in other tinnitus therapy programs were not included in the study [Tass et al., 2012a]. The extent of the hearing loss was investigated with a pure tone audiogram. Patients with a hearing loss at any of the tested frequencies (i.e., 0.125, 0.250, 0.750, 1, 2, 3, 4, 6, 8, 12 and kHz) >50 dB were not included in the study. By the same token, patients who were not able to hear all CR therapy tones (see below) were excluded from the study. In brief, 1–4 and 6–12 kHz pure‐tone averages (PTAs) were calculated.

From 63 randomized patients, 61 had EEGs recorded at baseline and 12 weeks. In all, 11 out of these 61 patients were excluded from the analysis as their EEG recordings were performed with lower hardware filter settings (0.1–50 Hz), not allowing for an analysis of higher *γ*‐band activity. Unilateral and bilateral tinnitus patients can have different EEG abnormalities [Vanneste et al., 2011a]. Accordingly, to avoid an influence of such differences, we selected only the patients with bilateral tinnitus (*n* = 28). An overview of the patient's baseline characteristics is summarized in Table 1.

**Table 1:** Baseline characteristics of all patients and the patients with a good clinical response (i.e., TQ improvement ≥ 12 points)

	All patients (*n* = 28)	Patients with a good clinical response (*n* = 12)
Age (years) (SD)	50.0 (10.5)	49.3 (8.9)
Tinnitus duration (years) (SD)	6.1 (4.7)	7.8 (5.6)
TQ (SD)	45.6 (16.8)	52.3 (17.5)
VAS‐L (SD)	68.4 (19.2)	72.1 (20.7)
VAS‐A (SD)	67.7 (20.5)	71.7 (23.1)
1–4 kHz pure‐tone average	14.83 (9.99)	14.42 (10.32)
6–12 kHz pure‐tone average	36.55 (17.65)	33.19 (13.29)

### CR Treatment

In the RESET study, patients were stimulated for 12 weeks using a portable acoustic device and comfortable earphones (for a more detailed description of the CR neuromodulaton treatment, see Tass et al., 2012a). Visits took place after 1, 4, 8, 12, and 16 weeks. Data for this article were obtained from the baseline and the 12‐week visit, because EEG recordings for all patients were performed off‐stimulation (i.e., at least 2.5 h after cessation of CR neuromodulation) at these visits. Subjectively perceived tinnitus loudness and tinnitus annoyance were assessed off‐stimulation using a visual analog scale for loudness (VAS‐L) and annoyance (VAS‐A). In general, the CR treatment resulted in a highly significant and clinically relevant decrease of tinnitus severity as measured by VAS‐L/VAS‐A and TQ scores [Adamchic et al., 2012a,2012b; Tass et al., 2012a].

### Healthy Controls

The control group consisted of 16 healthy tinnitus‐free subjects (10 men and 6 women) age matched (mean age 45.0, SD 12.5; *P* = 0.29) to the group of 28 tinnitus patients selected for the EEG analysis. Recruitment of study participants, included in the control group, was performed by advertisement and all participants signed informed consent form. Participants, selected to the control group, were screened by physicians for neurological and mental disorders as well as for ear disorders. Subjects taking CNS‐acting medication were excluded.

The PTAs of 1–4 and 6–12 kHz were 12.73 (13.69) and 24.29 (19.05), respectively. All healthy subjects were not taking any medication known to affect the EEG. This group was selected to confirm tinnitus‐related EEG abnormalities reported previously [De Ridder et al., 2011b; Moazami‐Goudarzi et al., 2010; Weisz et al., 2005]. Furthermore, the control group served as a reference for the treatment‐induced EEG changes.

### Patient Groups for EEG Analysis

We performed three different comparisons: (i) We compared all 28 patients with bilateral tinnitus before CR therapy with the healthy control group. (ii) We compared all 28 patients with bilateral tinnitus after 12 weeks of CR therapy with the healthy control group. (iii) To investigate symptom‐related changes in the oscillatory brain activity, we also investigated a subgroup of patients with a good clinical response defined as TQ improvement > 12 points (*n* = 12) as they were expected to display the most pronounced EEG changes. A TQ > 12 cut‐off was selected based on the Reliable Change Index [Jacobson and Truax, 1991] with the assumption that it separates patients with moderate to good relief of their tinnitus symptoms from patients with small to no relief in symptoms [Tass et al., 2012a; Turner et al., 2010].

The group of all 28 patients with bilateral tinnitus comprised patients from all therapy Groups G1–G4: 10 from G1, 4 from G2, 6 from G3, and 8 from G4. The group of good responders (*n* = 12) consisted of six patients from G1, four from G3, and two from G4.

### EEG Data Acquisition

#### Patients

Every patient underwent two recording sessions: first on the first treatment day before start of the treatment; second at the 12‐week visit, minimum 2 h after stopping the last stimulation session.

#### Healthy controls

In every healthy control subject, one EEG recording was performed. Patients and healthy controls were instructed to retain from caffeinated beverages on the day of the recording. Patients and controls were seated in an upright position in a comfortable chair. EEG recordings were obtained in a dimly lit room in a Faraday cage. EEG data were collected from 128 surface electrodes (128 channel HydroCel Geodesic Sensor Net). All electrodes were referenced to Cz. The EEG signals were amplified with a Net Amps 200 amplifier (Electrical Geodesis, Eugene, OR), digitized at 1 kHz and band‐pass filtered from 0.1 to 400 Hz. Recordings were performed during awake state with eyes closed and eyes open alternating 2‐min long conditions. We selected the eyes closed data (two 2‐min long epochs) for further analysis as they were less affected by artifacts. Subjects were video monitored for behavioral signs of drowsiness and the epochs with signs of drowsiness were excluded from further analysis. We were interested in spontaneous activity; therefore, we excluded the transitions between eyes‐open and eyes‐closed phases by removing the first and the last 5 s of each eyes‐closed epochs. Photogrammetry was performed for all subjects using the Geodesis Photogrammetry system (Electrical Geodesis, Eugene, OR) and the individual head shape was modeled for each subject and EEG session.

### Data Analysis

The scalp EEG was rereferenced to average reference. Signals were additionally digitally filtered with a 0.8–130 Hz digital filter. Each EEG recording was corrected for blink and eye movements in Brain Electrical Source Analysis (BESA) using the surrogate model approach in BESA (MEGIS Software, 5.2 version) [Ille et al., 2002]. Recordings were further analyzed in MATLAB (The Mathworks, Natick, MA) using EEGLAB (http://sccn.ucsd.edu/eeglab) where artifact rejection was performed. First, epochs containing large non‐neural artifacts (e.g., large EMG artifacts, movement artifacts) were removed in EEGLAB. Then, independent component analysis decomposition was performed on the sensor level EEG data [Delorme et al., 2007]. Myogenic components, that is, components containing EMG activity in the absence of any identifiable neurogenic activity, were selected. These components were identified based on the following criteria: (i) high broad peaks around either 30–40 Hz and higher, (ii) a moderately small and clustered distribution on the topographic maps that mimicked the underlying scalp musculature, (iii) periods of high‐frequency activation in the time domain, and (iv) equivalent dipole(s) located outside the brain volume and having a residual variance of 15% or less. Dipole locations were modeled using DIPFIT plug‐in in EEGLAB. Head shape and electrode locations were modeled for each subject and EEG session separately using the EGI Photogrammetry system and then imported into the DIPFIT plug‐in. Myogenic component selection procedure was performed twice with inter‐rater reliability assessed using Krippendorff's alpha (*α* = 0.97). The mean length of the recordings after artifact correction was 3 min 36 s ± 24 s. Surface EEG was transformed into brain source activity using the source montage approach in BESA [Scherg et al., 2002]. A source model was generated with regional neural sources placed in the regions of interest (ROIs) using BESA. The source montage consisted of temporal (T), orbitofrontal (OF), dorsolateral prefrontal cortex (DPFC), and parietal (PA) sources in both hemispheres, one source was also located within the anterior cingulate cortex (CA) and one in the posterior cingulated cortex (CP). We have to point out that the source montage approach used here should not be confused with a fine spatial localization of, for example ERP, activity performed in some functional neuroimaging studies for each patient and condition. Location of regional sources (ROIs) was predefined by the authors based on the results of the previous studies and was the same for every patient and recording [Lanting et al., 2009; Schlee et al., 2009a; Vanneste et al., 2011b; Weisz et al., 2005]. Additional probe‐sources were placed into the occipital lobe and in the area of the central sulcus in both hemispheres. Sources outside the ROIs acted as a spatial filter and reduced the contribution of these regions to the ROI. The strength of the source montages approach is that one can obtain time courses of brain activities from distinct brain regions [Scherg et al., 2002]. For the PLS statistical analysis, the temporal source was further subdivided into the two sources: (1) an equivalent dipole modeling the primary auditory cortex (ROI: AC1) with Thalairach coordinates [*x*,*y*,*z*; mm] ±40, −26, −12 and orientations: ±0.3, −0.5, −0.8 left and right [Verkindt et al., 1995]; (2) the secondary auditory cortex (AC2) was modeled with a dipole having a radial orientation [Hegerl et al., 1994]. Normalized band powers from 5 ROIs (AC1, AC2, OF, DPFC, and PA) within the left and right hemispheres were averaged over hemispheres for each ROI and every patient separately, resulting in seven ROIs for 28 patients [Kahlbrock and Weisz, 2008]. This averaging was justified by standardized low‐resolution brain electromagnetic tomography (sLORETA) statistical maps that showed a similar spatial distribution of statistically significant differences in both hemispheres. Furthermore, in tinnitus patients altered spontaneous activity pattern in the temporal region was found to be bilaterally symmetrical, and the same holds true also for activation during the acoustic stimulation paradigm [Smits et al., 2007; Weisz et al., 2005]. Thus, no unilateral effects were affected and/or masked through averaging over both hemispheres. The fast Fourier transform was performed on the artifact‐free source waveforms after windowing the signal with a 4,096 ms wide cosine squared (cos^2^) window with 50% overlap. This gave us a frequency resolution of 0.244 Hz.

The following frequency bands were defined: 1–3.5 Hz (*δ*), 4–7.5 Hz (*θ*), 8–12 Hz (*α*), 12.5–30 Hz (*β*), 30.5–48 Hz (low *γ*), and 52–90 Hz (high *γ*). In all calculations, we excluded the power line artifact (48–52 Hz). Taking into account the comparatively poor test–retest reliability for absolute as compared to relative power bands, we performed our analysis based on the relative power features [John et al., 1983]. The individual power spectra were normalized by dividing power at each frequency by the integral of the power across all frequencies from 1 to 90 Hz. This allowed us to compare subjects with large differences in the overall spectral energy and to estimate the relative contribution of each band to the whole spectrum and to compare the relative contribution of different bands. For the multivariate analysis, power spectra derived with BESA source montage analysis were divided into 1‐Hz wide bands in the range from 1 to 90 Hz.

As shown in Figures 5 and 6, for a better presentation, these 1‐Hz wide bands were labeled by abbreviations consisting of the lower edge of the frequency band followed by the ROI. For instance, 25DPFC stands for the frequency band between 25 and 26 Hz in the DPFC cortex. We used sLORETA to confirm the results received with BESA source montage [Pascual‐Marqui, 2002]. With sLORETA, we computed a three‐dimensional linear inverse solution to the EEG inverse problem with a three‐shell spherical head model registered to the Talairach human brain atlas digitized at the Brain Imaging Center of the Montreal Neurological Institute [Pascual‐Marqui, 2002]. The solution space was constrained to the gray matter voxels that belonged to cortical and hippocampal regions (a total of 6,430 voxels at a 5‐mm spatial resolution). The localization of the differences in current density power between the groups was assessed by voxel‐by‐voxel *t*‐tests of the sLORETA images. In the resulting statistical three‐dimensional maps, a nonparametric approach was used to identify statistically significant differences of cortical voxels [Nichols and Holmes, 2001]. Briefly, if there were no significant differences between groups, any labeling of voxels would result in an equally likely statistical map. Thus, sLORETA statistical maps were randomly relabeled and *t*‐values were recalculated. Under the null hypothesis, each of the *t*‐statistics are equally likely, and thus, the resulting *P*‐value is the proportion of the *t*‐statistic values greater than or equal to the *t*‐statistic of the correctly labeled data (for more details, see Nichols and Holmes, 2001). *P*‐values were derived from 5,000 such permutations. The voxels' *P*‐values of 0.05 were colored in a MRI template.

### Statistical Analysis of Spectra

For comparisons of power spectra between groups, a nonparametric Wilcoxon rank sum test for each frequency point was used. This statistical test for frequency point comparisons was corrected for the number of tests conducted using the false discovery rate (FDR, Benjamini and Hochberg, 1995).

### Multivariate Analysis

To determine the relationship between power spectra changes (calculated from BESA ROIs) and changes in clinical scores, we applied the PLS analysis [Krishnan et al., 2011]. Power changes in 1‐Hz‐wide frequency bands were used for multivariate analysis as predictor variables of changes in TQ scores and VAS values. PLS analysis was selected as it overcomes the problems related to multicollinearity and having too many variables as compared to the number of samples.

All 28 patients were included into the PLS model. The power spectra were divided into 1‐Hz wide bands starting at 1 to 90 Hz, which yielded a total of 85 1‐Hz wide bands. The power in each band was normalized to the range of 1–90 Hz total power. Changes from the first session to the second session were calculated for each 1‐Hz band and every patient separately. The power values for the multivariate analysis were structured in an *X*‐matrix with one row per subject (i.e., a total of 28 rows) and one column per 1‐Hz‐wide frequency band for each source (i.e., a total of 595 columns).

The VAS and TQ data were structured into the *Y*‐matrix in an analogous way. For this analysis, we have used VAS‐L and VAS‐A values as well as TQ total, psychological distress (PD), intrusiveness (*I*), auditory perceptional difficulties (*A*), and sleep disturbance (Si). We did not include emotional distress (*E*) and cognitive distress (*C*) subscales as they are already represented in PD (PD = *E* + *C*) and somatic complaints (So) as it is difficult to rule out the real origin of somatic complaints (i.e., the *Y*‐matrix consisted of 28 rows and 7 columns). Several dependent variables can be modeled at the same time using PLS allowing for a simpler interpretation of the results. However, if the dependent variables are reasonably independent, computing a single PLS model tends to have many components and to be difficult to interpret. In such a case, a separate modeling of clusters of correlated dependent variables (Ys) results in a set of simpler models with fewer dimensions. Hence, in the process of creating a PLS model one should start with a principal component analysis (PCA) of the matrix of dependent variables (i.e., *Y*‐matrix) to characterize patterns or “structure” in data [Wold et al., 2004]. If dependent variables cluster in groups in the PCA loading plot, separate PLS models should be considered for each cluster [Wold et al., 2004]. Accordingly, first we performed a PCA on the *Y*‐matrix [Jolliffe, 2002]. The purpose of the PCA analysis was to reveal latent structures existing in the data. This allowed us to identify data components that clustered together and may have a systematic relationship. Accordingly, separate models were needed for each PCA cluster. PCA and PLS techniques share a common basis. Both techniques define the underlying structure of the data (i.e., the latent variables) by projecting linear planes into multidimensional data [Kramer, 1998]. PLS has one important feature compared to PCA: PCA determines latent variables only in one matrix (i.e., predictors), whereas PLS takes into account both the independent (*X*) and the dependent (*Y*) variables to build a predictive model. In our case, the PLS approach translates into a predictive model for *Y* (changes in clinical scores) depending on *X* (changes in power spectra). Put otherwise, the model describes which components of the power spectra covary with changes in symptoms. Each latent variable will explain a part of the variance in the data. Typically, several components are needed to explain most of the variances in the data. Also, it is desirable to have a smaller number of latent variables, for the results to be more manageable and accountable. Taking this into account, the goal of the PLS regression modeling was to explain the greatest part of the variations in the data with the smallest number of components. PLS was used to model the relationship between the 595 normalized 1‐Hz wide power bands as predictive variables on the one hand and the groups of clinical test scores, revealed by PCA, as dependent variables on the other hand.

The goodness of fit of the model was evaluated in terms of both *R* ^2^ (which is analogous to the Pearson product–moment correlation coefficient) and the goodness of prediction *Q* ^2^ as determined by a crossvalidation procedure [Vandervoet, 1994; Wold, 1978]. Both *R* ^2^ and *Q* ^2^ were calculated for the original data as well as for 20 random permutations of the dependent variable matrix *Y*, whereas keeping the *X* matrix fix. As the matrix of dependent variables [*Y*_1_] changes to a new, reordered matrix [*Y_r_*], the correlation between these two matrices (corr[*Y*_1_,*Y*_r_]) decreases. If there is a nonrandom relationship between predictors and dependent variable, the predictive PLS model, constructed on the reordered data, should have a predictive power (*Q* ^2^) that decreases as the correlation between *Y*_1_ and *Y_r_* decreases. If this is not the case, and the PLS model for the reordered data shows a similar predictive ability (*Q* ^2^) as for the initial data, there is a good possibility for the model being based on chance.

We excluded less informative and redundant *X* variables (i.e., variables with low contribution for explaining the *Y* variable) from the modeling by the variable selection procedure [Marini et al., 2005]. For this purpose, the variable importance in the projection (VIP) was used that has been computed according to the formula used in the SIMCA‐P + 12.0.1 (http://www.umetrics.com/). Predictive variables that have a VIP value of >1 are most relevant for explaining dependent variables. Accordingly, all descriptors with a VIP value less than 1 were excluded from the model. This procedure was iteratively repeated until an optimal model (the one with the highest *Q* ^2^) was obtained.

## RESULTS

### Clinical Data

Mean improvement in the TQ and VAS‐L/VAS‐A scores for all 28 patients with bilateral tinnitus after 12 weeks of treatment was −11.0 ± 10.2 (*P* < 0.01) and −19.5 ± 22.6/−19.3 ± 21.6 (*P* < 0.01/*P* < 0.01), respectively. The subgroup of good responders (*n* = 12) showed a mean reduction of −19.5 ± 13.5 (*P* < 0.001) TQ points and −26.7 ± 26.2/−25.4 ± 24.0 (*P* < 0.01/*P* < 0.01) points in VAS‐L/VAS‐A. At 12 weeks, PTAs in the group of all tinnitus patients were 14.07 (10.25) for 1–4 kHz and 36.56 (17.80) for 6–12 kHz. In the group of good responders at 12 weeks, PTAs were 13.08 (9.25) for 1–4 kHz and 31.32 (15.21) for 6–12 kHz. No significant differences were found between baseline and the 12‐week visit PTAs in the tinnitus patients. Significant differences (i.e., *P* < 0.05) in the hearing thresholds (PTA, 6–12 kHz) were found between all tinnitus patients (*n* = 28; both at baseline and at 12 weeks) and the control group.

### Altered EEG Rhythmicity

#### Mean power spectra

**Figure 1:**
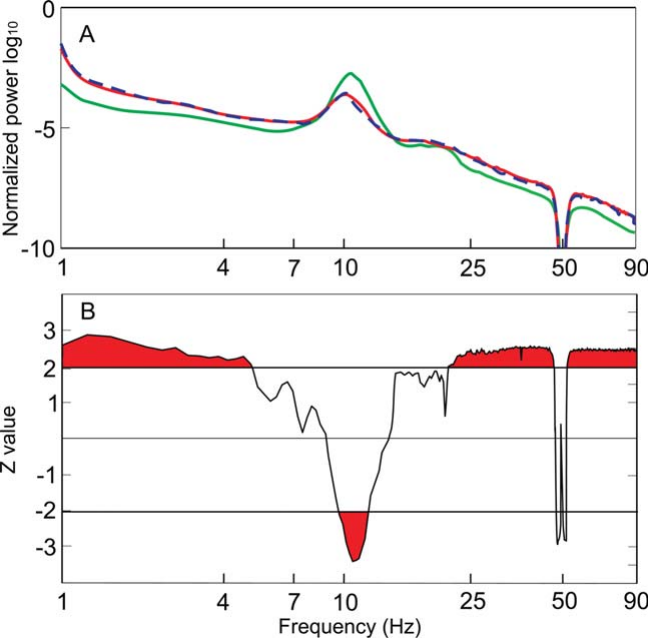
Enhanced *δ* and *γ* EEG power in tinnitus patients. **A**: In the global EEG power spectrum, *δ* and *γ* power were increased and *α* power was decreased in tinnitus patients recorded before CR neuromodulation (*n* = 28, red line) as compared to the healthy controls (*n* = 16, green line). In contrast, the global EEG power spectrum of the group of good responders (*n* = 12, blue dashed line) did not differ from that of the group of all patients with bilateral tinnitus before CR therapy. **B**: Areas indicated with red (i.e., above or below the horizontal line) correspond to statistically significant differences between all bilateral tinnitus patients (*n* = 28) and the healthy controls. Significant differences were found in *δ*, low *θ*, *α*, high *β*, low and high *γ* bands.

Let us first consider the EEG at baseline, that is, prior to CR therapy. To summarize the data from all EEG scalp sensors and all subjects, we averaged the individual spectra from all tinnitus patients recorded before CR neuromodulation (*n* = 28), from the subgroup of good responders (*n* = 12) and from the healthy controls (*n* = 16), respectively (Fig. 1). The average power spectrum in the group of all bilateral tinnitus patients before CR therapy (*n* = 28) showed clear differences from the average power spectrum of the control group. In the patients group, the average spectral power was higher in *δ* (1–3.5 Hz), low *θ* (≤5 Hz), high *β* (≥20 Hz), low *γ* (30.5–48 Hz), and high *γ* (52–90 Hz) bands. In contrast, the spectral power in the high *α* band (9.7–12 Hz) was lower in the tinnitus patients group. In the tinnitus population, the *α* peak frequency (i.e., the frequency at which the *α* peak was located) prior to CR therapy was shifted toward lower frequencies as compared to the healthy controls; however, this difference was not significant (*P* = 0.11). The subgroup of 12 good responders (Fig. 1A, blue dashed line) showed the same differences from the healthy population in all frequency ranges as the complete group of 28 bilateral patients before therapy (Fig. 1A, red line).

#### BESA source montage analysis

**Figure 2:**
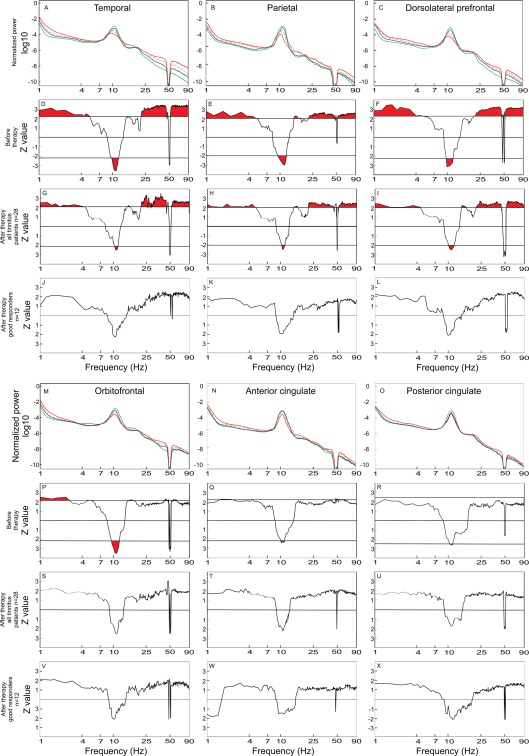
Power spectra for the group of healthy controls (*n* = 16) (green line), the group of all bilateral tinnitus patients (*n* = 28) before therapy (red line), the group of all bilateral tinnitus patients (*n* = 28) after therapy (orange dashed line), and the subgroup of good responders (*n* = 12) (blue line) in the temporal (**A**), PA (**B**) and DPFC (**C**), OF (**M**), anterior cingulated (**N**), and posterior cingulated (**O**) ROIs. The power spectra of all 28 bilateral tinnitus patients before therapy were compared to those of the healthy controls (*n* = 16) by means of the Mann–Whitney *U*‐test in the temporal (**D**), PA (**E**) and DPFC (**F**), OF (**P**), anterior cingulated (**Q**), and posterior cingulated (**R**) ROIs for each frequency point. In the same manner, the power spectra of all 28 bilateral patients after therapy were compared to the spectra of the healthy controls (*n* = 16) (**G**, **H**, **I**, **S**, **T**, **U**). The analogous comparison was also performed between the good responders (*n* = 12) and the healthy controls (*n* = 16) (**J**, **K**, **L**, **V**, **W**, **X**) in the temporal, PA, DPFC, OF, anterior cingulated, and posterior cingulated ROIs, respectively. Areas indicated with red (i.e., above or below the horizontal line) correspond to statistically significant differences. In plots with neither red areas nor horizontal significance lines, no frequency point attained significance threshold. There is a noticeable trend toward a reduced *α* peak frequency in the tinnitus population. Supporting Information Figure S1 shows topographical plots of EEG power differences in the specific frequency bands.

Furthermore, we studied which brain regions contributed most to the significant differences between patient and control group and at which frequencies. Mann–Whitney–*U*–tests were performed for each regional source at each frequency point (Fig. 2). The maximal *Z*‐value (|*Z*| = 3.6) was observed in the 8–12 Hz *α* band in the temporal regions. *Z*‐values in the temporal region were highest compared to the other ROIs in most of the frequency bands. The subgroup of 12 patients with a TQ improvement of ≥12 showed no significant differences in spectral power to the subgroup of 16 patients with a TQ improvement of <12 in any of the ROIs: temporal (*F* = 0.79, *P* = 0.59), PA (*F* = 0.75, *P* = 0.62), DPFC (*F* = 0.54, *P* = 0.77), OF (*F* = 0.88, *P* = 0.52), anterior cingulate (*F* = 1.01, *P* = 0.45), and posterior cingulate (*F* = 1.53, *P* = 0.22). We also investigated possible effects of tinnitus duration in patients with a tinnitus history of ≤4 years and >4 years, respectively [Schlee et al., 2009a]. There were no significant differences in spectral power between patients with tinnitus duration of ≤4 years and patients with tinnitus duration of >4 years in any ROIs: temporal (*F* = 0.57, *P* = 0.75), PA (*F* = 0.62, *P* = 0.71), DPFC (*F* = 0.69, *P* = 0.66), OF (*F* = 0.65, *P* = 0.69), anterior cingulate (*F* = 0.75, *P* = 0.62), and posterior cingulate (*F* = 0.66, *P* = 0.68).

#### Standardized low‐resolution brain electromagnetic tomography

**Figure 3:**
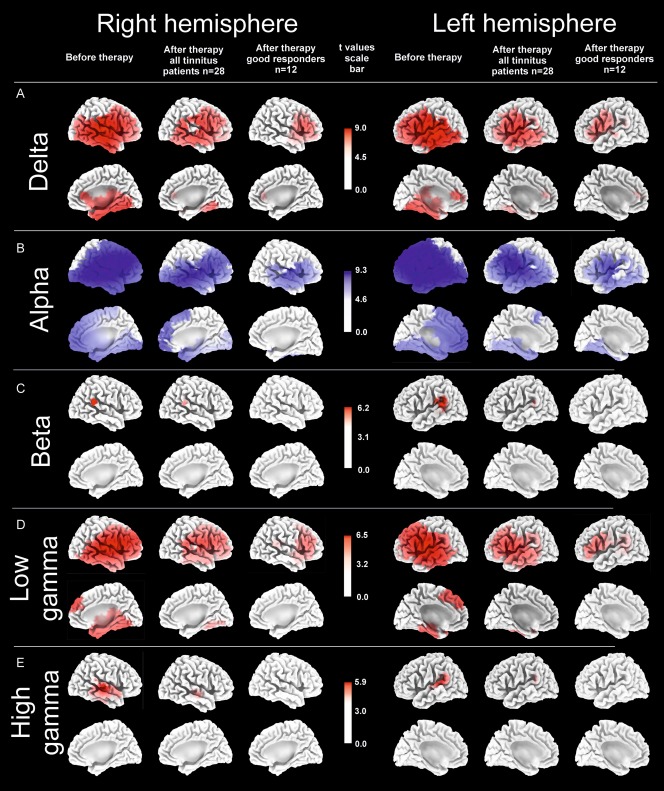
sLORETA functional tomographic maps of the significant differences in the power of regional electric brain activity between all 28 bilateral tinnitus patients before therapy and healthy controls (“Before therapy”), between all 28 bilateral tinnitus patients after 12 weeks of therapy and healthy controls (“After therapy all tinnitus patients *n* = 28”) and between 12 good responders after 12 weeks of therapy and healthy controls (“After therapy good responders *n* = 12”) in five EEG frequency bands *δ* (1–3.5 Hz), *α* (8–12 Hz), *β* (12.5–30 Hz), low *γ* (30.5–48 Hz), and high *γ* (52–90 Hz). In the *θ* band (4–7.5 Hz) no significant differences were found between tinnitus patients and healthy controls, neither before nor after CR therapy. Voxels of significantly decreased power (*P* = 0.05) in tinnitus patients as compared to healthy controls are labeled in blue, whereas voxels with significantly increased power (*P* = 0.05) are labeled in red. After 12 weeks of acoustic CR neuromodulation, there was a pronounced decrease of number of voxels with power that was significantly different from the healthy controls: In the *δ*, *β* as well as low and high *γ* band initially pathologically enhanced power decreased, whereas in the *α* band the initially reduced power reincreased.

sLORETA images show significant power differences in different frequency bands between tinnitus patients and healthy controls (Fig. 3). As opposed to the healthy controls, the cortical spectral power of all bilateral tinnitus patients before the start of the treatment was significantly enhanced in the *δ*, *β*, low *γ*, and high *γ* frequency band. In contrast, in the *α* band, we observed a reduced cortical spectral power in the tinnitus population as compared to the healthy controls. The reduced *α* power was found in prefrontal dorsolateral and medial, anterior and posterior cingulate, orbitofrontal, parietal and temporal, and other regions (Fig. 2 and Table 2).

**Table 2:** Statistically significant results from the comparison of the group of all bilateral tinnitus patients after 12 weeks of therapy (*n* = 28) with healthy controls and the group of good responders after 12 weeks of therapy (*n* = 12) with healthy controls^a^

*δ* Left	*δ* Right
1, 2, 3, 4, 6, 8, 9, 10, 11, 13, 18, 19, 20, 21, 22, 23, 24, 27, 28, 29, 30, 32, 34, 35, 36, 37, 38, 39, 40, 41, 42, 43, 44, 45, 46, 47	1, 2, 3, 4, 6, 8, 9, 10, 11, 13, 17, 18, 19, 20, 21, 22, 23, 24, 25, 27, 28, 29, 30, 32, 33, 34, 35, 36, 37, 38, 39, 40, 41, 42, 43, 44, 45, 46, 47
*α* Left	*α* Right
1, 2, 3, 4, 6, 8, 9, 10, 11, 13, 17, 18, 19, 20, 21, 22, 23, 24, 25, 27, 28, 29, 30, 31, 32, 33, 34, 35, 36, 37, 38, 39, 40, 41, 42, 43, 44, 45, 46, 47	1, 2, 3, 4, 5, 6, 8, 9, 10, 11, 13, 17, 18, 19, 20, 21, 22, 23, 24, 25, 27, 28, 29, 30, 31, 32, 33, 34, 35, 36, 37, 38, 39, 40, 41, 42, 43, 44, 45, 46, 47
*β* Left	*β* Right
13, 22, 40, 41, 42	22, 40, 42
Low *γ* left	Low *γ* right
1, 2, 3, 4, 6, 8, 9, 10, 11, 13, 19, 20, 21, 22, 27, 28, 32, 34, 35, 36, 37, 38, 39, 40, 41, 42, 43, 44, 45, 46, 47	1, 2, 3, 4, 6, 8, 9, 10, 11, 13, 18, 19, 20, 21, 22, 23, 25, 27, 28, 29, 30, 32, 34, 35, 36, 37, 38, 40, 41, 42, 43, 44, 45, 46, 47
High *γ* left 13, 22, 40, 41, 42	High *γ* right 13, 20, 21, 22, 38, 41, 42

a BAs comprising at least one significant voxel were included in the table as areas showing significant power differences. Areas not listed here did not show significant power differences between the compared groups. BAs printed in black exhibited significant differences in the group of all bilateral tinnitus patients (before and after therapy) as well as in the group of good responders as compared to the healthy controls, respectively. The power in the black BA was, hence, significantly different from the healthy controls and was not normalized during therapy. BA printed in red were significantly different in the bilateral tinnitus population (*n* = 28) from the healthy controls only before the start of the therapy. The power in the red BAs was, thus, significantly normalized in all bilateral patients. BAs printed in blue were significantly different in the group of all bilateral tinnitus patients (*n* = 28) from the healthy controls both before and after the therapy, but lost their significance in the group of good responders after 12 weeks of therapy (*n* = 12). Hence, the power in the blue BAs was normalized after 12 weeks of therapy in the group of good responders only.

Spatial peaks (i.e., local maxima) of the spectral power prior to the start of the therapy were localized in the temporal cortex in the *δ* band (Brodman area [BA] 42, *t* = 8.98) and in the temporal and DPFC in the low *γ* band (BA 41, *t* = 6.43; BA 46, *t* = 6.36). Spatial troughs (i.e., local minima) of the spectral power were found in the *α* band in the temporal (BA 22, *t* = 9.16) and in the DPFC (BA 46, *t* = 9.23). No significant differences between tinnitus patients and healthy controls were found in the *θ* frequency range.

### Treatment‐Induced Changes

Furthermore, we studied the effect of acoustic CR neuromodulation on the spontaneous oscillatory brain activity. To this end, first, we investigated the group of all patients with bilateral tinnitus after 12 weeks of CR treatment. The power spectrum of this patient group approached the average spectrum of the healthy control group, with the most prominent changes being observed in the temporal regions (Fig. 2). Consequently, we observed a decrease of the *Z*‐values, indicative of significant differences between tinnitus patients and healthy controls, in a wide range of frequencies (*δ*, *α*, low and high *γ*) after 12 weeks of CR therapy (Fig. 2). Further, we investigated a group of good responders (*n* = 12), that is, patients with a pronounced reduction of their tinnitus symptoms: TQ reduction ≥12 points. The average EEG spectrum of these patients approached the average spectrum of the healthy controls to an even greater extent than that of all 28 bilateral tinnitus patients and, accordingly, resulted in lower *Z*‐values for *δ*, *α*, low and high *γ* bands and disappearance of significant differences in *θ* and *β* bands (Fig. 2). No significant differences were found in any of the ROIs in the group of 12 good responders after 12 weeks of therapy (Fig. 2).

Statistical sLORETA images reveal a reduction of the number of voxels and, consequently, the number of BAs displaying a significantly different power between all bilateral tinnitus patients after therapy and healthy controls (Fig. 3 and Table 2). The number of voxels with power values that significantly differed from those of the healthy population was reduced in the group of all 28 tinnitus patients (Table 3). To an even greater extent, the number of voxels with significant power differences decreased in the group of good responders after 12 weeks of CR treatment (Table 3).

**Table 3:** Reduction of number of voxels showing a significant power difference compared to the healthy controls

	All 28 patients (%)	12 good responders (%)
*δ*	−42.6	−77.4
*α*	−31.1	−68.3
*β*	−44.9	−100.0
Low *γ*	−44.4	−78.8
High *γ*	−83.4	−100.0

Interestingly, spatial peaks (i.e., local maxima) of the spectral power in the group of all bilateral tinnitus patients after 12 weeks of therapy were still localized in the temporal cortex in the *δ* (BA 41, *t* = 7.53) and low *γ* (BA 41, *t* = 5.24) band. In contrast, in the group of 12 good responders the spatial peaks (i.e., local maxima) of the spectral power were located in the frontal lobe for *δ* (BA 44, *t* = 5.67) and low *γ* (BA 46, *t* = 4.32), whereas the temporal lobes lost nearly all of their significantly different voxels after 12 weeks of CR therapy (Fig. 3). Remarkably, no significant differences were found after treatment in the group of good responders in the *β* and high *γ* frequency band anymore. A list of BAs displaying significant results according to the sLORETA comparison between tinnitus patients versus healthy controls is summarized in the Table 2.

### Association of Changes in Spontaneous Brain Activity with Reduction of Tinnitus Symptoms

#### Principal component analysis

**Figure 4:**
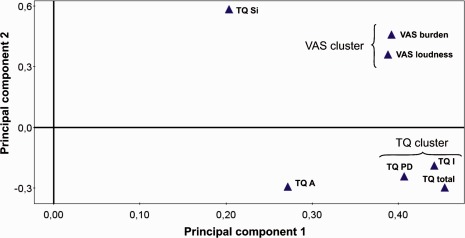
Loading plot showing the grouping of the clinical scores. Items grouped together responded in a similar way to the therapy, and their proximity reflects the strength of the similarity of their responses. The separation of TQ and VAS scores in distinct clusters suggests that they responded differently to the therapy. TQ subscores: TQ PD (psychological distress), TQ I (intrusiveness), TQ *A* (auditory perceptual difficulties), and TQ Si (sleep disturbances).

The loading plot in Figure 4 shows the results of the PCA on the changes of the VAS and TQ scores. The loading plot shows a clustering of the TQ and VAS according to the similarity of their responses to the therapy. The TQ PD and TQ I (intrusiveness) subscores and the TQ total score turn out to cluster together, whereas VAS‐L and VAS‐A form another and separate cluster. The TQ *A* (auditory perceptual difficulties) and TQ Si (sleep disturbances) subscores were both located at different and, in particular, remote sites compared to the two clusters mentioned above. This indicates that changes in TQ PD and TQ I subscores and the TQ total score on the one hand and VAS‐L and VAS‐A on the other hand as well as TQ *A* and TQ Si subscores, respectively, responded differently to the therapy and might be associated with different underlying mechanisms or were connected to similar mechanisms but to a different extent. Based on these results, a further PLS regression modeling was performed, TQ PD and TQ *I* subscores as well as TQ total scores were analyzed separately from TQ *A* and TQ Si subscores, being further separated from VAS‐L and VAS‐A.

#### Partial least‐squares

**Figure 5:**
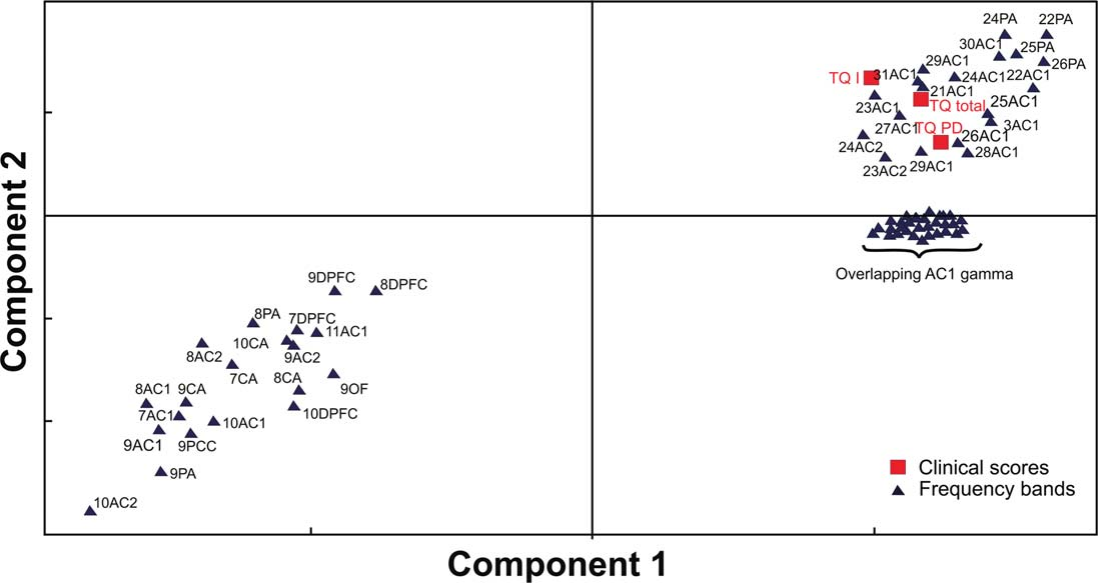
Loading plot showing the changes of TQ scores and spectral power bands for the first and second PLS component. The loading plot shows the 70 1‐Hz wide power bins and the TQ PD and TQ I subscores together with the TQ total scores. The proximity of the changes in spectral power values and the TQ (sub‐)scores reflects the strength of the association of these changes to the changes in the clinical scores. The 1‐Hz‐wide frequency bands were labeled by abbreviations consisting of the lower edge of the frequency band followed by the ROI (for abbreviations, see **METHODS** section). ROI abbreviations: primary auditory cortex (AC1), secondary auditory cortex (AC2), orbito‐frontal (OF), dorsolateral‐prefrontal (DPFC), parietal (PA), anterior cingulate cortex (CA) and posterior cingulated cortex (CP).

First, we performed the PLS for the spectral power variables versus three TQ‐dependent variables (TQ PD, TQ *I* subscores, and TQ total scores). This resulted in a PLS model with good performance (*R* ^2^ = 0.66, *Q* ^2^ = 0.4). A model validation by permutations indicated the presence of nonrandom associations in the model. The strength of the associations between EEG and TQ scores was determined according to a loading plot (Fig. 5).

This loading plot shows the clustering of the predictor variables and the dependent variables, indicating the strength of the association (covariance) between changes in neuronal power with changes in the individual TQ scores. The loading plot combines both PLS components included into the model. The widest positive association was found between the changes in TQ scores and the changes in AC1 *β*, low and high *γ* activity. Numerous frequency bands in other sources were also positively associated with the changes in symptoms as assessed by the TQ (sub‐)scores (Fig. 5). Other frequency bands (located in the left lower quadrant of Fig. 5) were negatively associated with the TQ PD and TQ *I* subscores and the TQ total scores.

**Figure 6:**
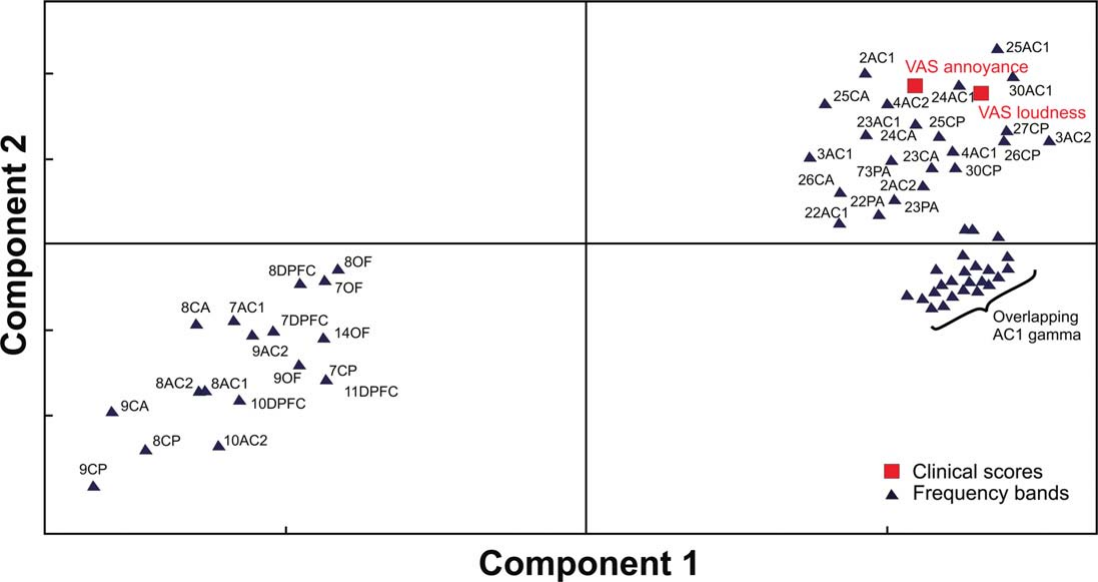
Loading plot showing the 67 1‐Hz wide power bands and VAS loudness and VAS annoyance scores. Proximity of the changes in the power values across different frequency bands to the changes in VAS reflects the strength of association of these neuronal changes to the changes in the clinical scores. For ROI abbreviations, see **METHODS** section and Figure 5.

A similar model performance was obtained for the PLS model with respect to changes of the VAS‐L and VAS‐A scores. Two components with 67 predictors resulted in a model with good performance (*R* ^2^ = 0.65, *Q* ^2^ = 0.41). Figure 6 shows the loading plot with the VAS‐L and VAS‐A as dependent variables and the frequency bands as predictor variables.

The most pronounced positive associations with the changes in the VAS were found for the changes in the following sources and frequency bands: ACI and ACII high *δ*/low *θ*, *β*, low and high *γ*. Frequency bands in the left bottom corner of the Figure 6 were negatively associated with the VAS‐L and VAS‐A. Interestingly, in the PLS model for the VAS scores, as opposed to the PLS model for the TQ scores, we found a higher influence of limbic areas (CA and CP) on the clinical VAS scores (Fig. 6). No satisfactory PLS models were found for TQ *A* and TQ Si subscores. No significant correlations were found between hearing thresholds (PTAs) and power in any of the frequency bands.

## DISCUSSION

We investigated oscillatory brain activity in patients with chronic subjective tonal bilateral tinnitus before and after 12 weeks of treatment with acoustic CR neuromodulation in comparison to healthy controls. To crosscheck our results, we used two qualitatively different inverse techniques: BESA source montage analysis [Scherg et al., 2002] and current source density reconstruction by sLORETA [Pascual‐Marqui, 2002]. Overall, we found that tinnitus patients significantly deviated from the healthy controls, concerning their oscillatory brain activity. Furthermore, CR therapy significantly normalized the patients' brain oscillations in all frequency bands tested, even leading to a complete abolishment of pathological power in several brain regions and frequency bands in the good responders. For instance, based on the BESA source montage analysis [Scherg et al., 2002], the abnormal power alterations completely vanished in the good responders over the entire frequency range tested (i.e., from 1 to 90 Hz) in both the PA source and the cingulate source (Fig. 2). With the spatially more refined sLORETA analysis [Pascual‐Marqui, 2002], we revealed that in the good responders pathologically enhanced *β* and high *γ* power vanished in the entire cortex, whereas *δ* and low *γ* decreased (nearly) completely in temporal and PA regions, whereas *α* power undergoes a widespread restitution (Fig. 3). In addition, in the patient group, the dominant *α* peak tended to be shifted to lower frequencies compared to the healthy controls.

Our results obtained with the two different inverse techniques, BESA source montage analysis [Scherg et al., 2002] and sLORETA analysis [Pascual‐Marqui, 2002], are in good agreement. However, in contrast to the BESA analysis, the sLORETA analysis did not reveal any statistical differences in *θ* band between healthy controls and tinnitus patients. This might be owing to the methodological differences between two methods: (1) Different resolution in the frequency domain: In contrast to our BESA analysis, where single‐frequency bins were investigated, our sLORETA analysis was performed on frequency bands of a width of several hertz. (2) Different statistical methods employed: In our BESA analysis FDR correction for multiple comparisons was performed for each frequency point [Benjamini and Hochberg, 1995]. For the sLORETA analysis, a voxel‐by‐voxel comparison was fed into a nonparametric test for functional brain imaging that corrects for multiple comparisons [Nichols and Holmes, 2001]. (3) Different technical features inherent to the different nature of the two inverse techniques, BESA [Scherg et al., 2002] being a source montage analysis as opposed to the distributed current density reconstruction by sLORETA [Pascual‐Marqui, 2002]. By the same token, changes of the *θ* power, found in our previous study by comparing EEG recordings in good responders pre‐ versus post‐CR treatment [Tass et al., 2012a], might not have been detected by the sLORETA analysis used in this study owing to fundamental methodological differences between the two approaches: In our previous study [Tass et al., 2012a], power changes of brain oscillations after CR neuromodulation were investigated in a paired groups design. In contrast, in this study, we used an independent group design that allowed us to compare tinnitus patients with healthy controls combining a cross‐sectional and a longitudinal approach. On the one hand, our results are relevant from a clinical standpoint. They show electrophysiological effects of acoustic CR neuromodulation and help to assess and possibly to further improve our therapeutic approach. On the other hand, a clinically successful intervention enables to elucidate the pathophysiology of tinnitus, in particular, the electrophysiological correlate of tinnitus (see also the discussion by Langguth et al. (2012)).

### Electrophysiological Characteristics of Tinnitus Patients

The enhanced synchrony at low and high frequencies combined with the decrease of *α* power (Figs. 2 and 3) is consistent with the previous findings and hence further indicates that this electrophysiological pattern to be a hallmark characteristic underlying chronic subjective tinnitus. The increased power at lower and higher frequencies in our patients is in line with the previous studies, reporting similar spectral abnormalities in individuals with tinnitus [Adjamian et al., 2012; De Ridder et al., 2011b; Weisz et al., 2005]. Our results are at variance with a study that revealed increased power in the frequency range of 2–100 Hz under eyes‐closed and eyes‐open condition in tinnitus patients as opposed to healthy controls [Moazami‐Goudarzi et al., 2010]. This might be owing to: (1) Different characteristics of the analyzed groups (e.g., groups size and type of tinnitus); (2) Different methods used to normalize power spectra.

In our data, the reduced *α* power was not caused artificially by the normalization, because in the group of tinnitus patients the *α* peak is much less prominent, wider, and shifted toward lower frequencies. Furthermore, we observe a similar spectral pattern also without the normalization. Our results are also in line with the study by De Ridder et al. [2011b] who described a case of a tinnitus patient with local field potential recordings via epicortical electrodes from the secondary auditory cortex. The patient was recorded in states with loud as well as little to no tinnitus. Decreased *α* power and increased power at lower frequencies were observed only in the presence of strong tinnitus [De Ridder et al., 2011b].

Increased power in spontaneous electrical brain activity was found in chronic tinnitus patients as compared to tinnitus patients with recent onset in *θ*, *β*, and *γ* bands [Vanneste et al., 2011b]. The chronic tinnitus patients also displayed a different distribution of the *γ* network [Schlee et al., 2009a]. However, in our study, there were no significant differences in oscillatory brain activity between patients with recent tinnitus onset (≤4 years) and patients with a long history of tinnitus (>4 years). These differences may be owing to different patient selection criteria or the choice of specific frequency band boundaries selected for statistical analysis. We may, thus, assume that the majority of the observed EEG changes in our study are related to the chronic tonal tinnitus, in general; however, we do not exclude the possibility of a partial effect owing to the tinnitus duration.

In our study, in the *δ* and low *γ* bands, that is, the bands that showed the most prominent and widest enhancement, the cortical generators of excess EEG power were predominantly located in temporal and frontal areas with spatial peaks of the power localized in the temporal cortex in the *δ* band (BA 42) and in the temporal and DPFCs in the low *γ* band (BA 41, 46). We found the foci of altered spontaneous oscillatory activity in tinnitus patients versus controls to be bilaterally located in temporal areas (see above), which resembles the previous findings [Weisz et al., 2005]. Concerning the differences between the symmetric bilateral distribution in both frontal lobes found here, as opposed to the left‐ or right‐sided differences reported in the previous studies, one has to take into account the relatively large and homogeneous group of tinnitus patients investigated in our study (i.e., 28 patients with chronic bilateral tonal tinnitus) in contrast to the smaller number of patients and/or mixed groups of patients with respect to laterality (i.e., bilateral vs. unilateral tinnitus) investigated in the previous studies [Moazami‐Goudarzi et al., 2010; Weisz et al., 2005]. Laterality of tinnitus has been previously reported to affect the pattern of spontaneous brain activity [van der Loo et al., 2009; Vanneste et al., 2011a]. Accordingly, confinement of the analyses presented in this manuscript to patients with bilateral tinnitus was done to reduce the heterogeneity which is present in tinnitus samples. Furthermore, our finding supports the notion of Weisz et al. [2005], who hypothesized that the asymmetry between hemispheres vanishes in case groups were used for analysis that are more balanced with respect to tinnitus side. Future studies will be necessary to investigate if these findings can be generalized to patients with unilateral tinnitus.

### Hearing Impairment

In our study, the tinnitus group had elevated auditory thresholds in the high‐frequency range compared to the control group. However, no significant changes in the hearing thresholds were detected in the tinnitus patients in the pre‐ versus post‐treatment condition. This is relevant in the following context. A major limitation of the pioneering MEG study by Weisz et al. [2005] was the significant difference in the status of hearing between tinnitus patients and healthy controls. That study revealed a pattern of brain rhythms characteristic of subjective tinnitus (enhanced *δ* and decreased *α*). However, the causal specificity of these findings remained a matter of debate, because in contrast to the normally hearing controls the tinnitus group had a high‐frequency hearing loss [Weisz et al., 2005]. Accordingly, it was not clear whether the observed pattern of brain rhythms had to be assigned to the tinnitus as opposed to the hearing impairment‐induced sensory deprivation [Weisz et al., 2005]. The latter might give rise to similar changes of brain activity as observed during slow‐wave sleep, where brain regions of increased *δ* and decreased *α* spatially coincide [Benoit et al., 2000]. However, in a previous study [Tass et al., 2012a] in one group of patients with tinnitus and after significant CR‐induced tinnitus relief, having the same hearing levels before and after therapy, we confirmed the characteristic tinnitus‐related pattern of brain rhythms as reported earlier by Weisz et al. (2005, 2007b). This fact underlines that the tinnitus‐related changes of brain rhythms, that is, enhanced *δ* and *γ* combined with decreased *α* [Weisz et al., 2005, 2007b], are tinnitus‐specific alterations [Tass et al., 2012a]. This notion is supported by the findings of Adjamian et al. (2012), who reported that increased *δ* activity in tinnitus patients was likely associated with tinnitus itself rather than deafferentation alone. Furthermore, this result is in accordance, for example, with the findings obtained in a study comparing spontaneous EEG in tinnitus patients with and without transitory changes of tinnitus loudness caused by residual inhibition [Kahlbrock and Weisz, 2008]. It is important to note that the EEG recordings analyzed in this article were obtained in our previous study mentioned above [Tass et al., 2012a].

### Thalamocortical Dysrhythmia

Our findings are in line with the concept of the thalamocortical dysrhythmia (TCD) [Llinas et al., 1999; Weisz et al., 2007a], that is, a mechanism characterized by a deafferentation‐induced increase of thalamic and, in turn, cortical low‐frequency oscillations, combined with cortical high‐frequency oscillations. Along the lines of this concept, slow‐wave oscillations are assumed to facilitate and sustain *γ* oscillations through a mechanism called the “edge effect” [Llinas et al., 1999, 2005]. According to the TCD concept, the enhanced synchronization in the *γ* range in temporal regions (BA 41) may be regarded as a direct electrophysiological correlate of the auditory phantom sensation [De Ridder et al., 2011b; Llinas et al., 1999; Weisz et al., 2007b]. As an indirect evidence of the validity of the TCD concept and the “edge effect,” in our tinnitus patients we observed enhanced synchronization in low‐ and high‐frequency bands in spatially greatly overlapping regions of the cortex (Fig. 3). The importance of *γ* oscillations for acoustic perception is well documented for healthy subjects [Crone et al., 2001] as well as for tinnitus patients [De Ridder et al., 2011b; van der Loo et al., 2009; Weisz et al., 2007b]. However, the emergence of enhanced *δ* and *γ* oscillations in frontal areas underlines the dysrhythmic coinvolvement of associative areas in the pathogenesis of tinnitus and may directly be related to the appearance of tinnitus‐related affective manifestations. The involvement of associative areas in relation to emotional factors in tinnitus has previously been reported in several studies [De Ridder et al., 2011a; Jastreboff, 1990; Lockwood et al., 1998; Mirz et al., 2000]. Additional evidence for the importance of associative areas for a conscious phantom percept comes from recordings from the somatosensory and the auditory system in nonhuman primates, which indicate that an activation of the primary sensory cortex is not sufficient for the generation of a conscious percept and implicate an involvement of the association cortex in conscious perception [Lemus et al., 2009a,2009b]. Accordingly, Jastreboff considered the prefrontal cortex as a “candidate for the integration of sensory and emotional aspects of tinnitus” [Jastreboff, 1990]. The relevance of a cognitive–emotional network in tinnitus was also convincingly pointed out by recent studies, demonstrating abnormal long‐range coupling in tinnitus patients [Schlee et al., 2009a, 2009b; Vanneste et al., 2011b]. Although the TCD concept [Llinas and Steriade, 2006; Llinas et al., 2005, 1999] underlines the importance of pathological low‐ and high‐frequency oscillations, the impact of reduced *α* oscillations should not be neglected [Weisz et al., 2007a]. Low levels of *α* are associated with a state of excitation, whereas high levels of *α* are associated with a state of inhibition [Klimesch et al., 2007; Weisz et al., 2007a].

### Effect of CR Neuromodulation

Both groups of tinnitus patients, that is, all bilateral tinnitus patients (*n* = 28) and the subgroup of good responders (*n* = 12) underwent a significant reduction of tinnitus symptoms (**RESULTS**). In general, acoustic CR neuromodulation reversed the increased cortical synchronization at low (*δ* and low *θ*) and high (*β*, low and high *γ*) frequencies, observed in individuals with tinnitus at baseline, toward physiological levels observed in the tinnitus‐free control group. This finding, combined with the significant covariance of changes in tinnitus symptoms and changes in EEG characteristics, provides further support for the hypothesis that tinnitus has an electrophysiological correlate as put forward previously [Llinas et al., 1999; Weisz et al., 2005].

Our results show that the activity in the slow and fast EEG frequency bands was decreased and in the *α* band increased after 12 weeks of CR neuromodulation in both groups with more prominent changes observed in patients with greater tinnitus reduction. There was a significant covariance between the degree of synchronization change in 1‐Hz‐wide frequency bands and the degree of tinnitus symptoms change that was strongest in temporal regions. This is consistent with the previous findings, demonstrating the leading role of the temporal cortex in coding tinnitus [De Ridder et al., 2011b; van der Loo et al., 2009; Weisz et al., 2007b], especially in its early phase [Schlee et al., 2009a]. However, the function of the primary sensory cortices is mainly to generate an appropriate neural representation of sensory input that by itself does not lead to conscious perception [Lemus et al., 2009a]. In fact, it requires the involvement of frontal and parietal areas, to make such a perception become conscious [De Ridder et al., 2011a; Lemus et al., 2009b]. Strong covariance of abnormal neuronal synchrony in the dorsolateral prefrontal regions with changes of tinnitus distress (assessed by the TQ), as revealed by PLS analysis, suggests that the frontal cortex is involved in a tinnitus‐related cortical network and is more associated with the affective distress of tinnitus. Analogously, abnormal neuronal synchrony in temporal regions is strongly associated with perceptual issues (i.e., aspects concerning the loudness and character of the sound).

Interestingly, we observed a higher covariance of the changes in limbic areas with changes in VAS rather than with changes in TQ scores (Figs. 5 and 6). Why changes in oscillatory brain activity in limbic areas were more strongly associated with VAS scores, but not with TQ scores? TQ covers a relatively wide spectrum of symptoms related to and caused by tinnitus, referring to a longer time interval. In contrast, to a certain extent, VAS is an emotionally more biased instrument of tinnitus assessment. Thus, the VAS scores might be more strongly influenced by the momentary emotional state of the patient and, consequently, be more associated with the oscillatory activity in the limbic areas present during the VAS assessment. Previous studies indicate that tinnitus intensity depends on the extent of abnormal neuronal synchrony in a distributed neuronal network, rather than on local *γ* oscillations [Schlee et al., 2009a; Vanneste et al., 2011b]. In line with this notion, our results show that acoustic CR neuromodulation causes a long‐lasting and widespread decrease of the power of both slow‐ and fast‐brain oscillations in a large network comprising auditory and nonauditory areas. In fact, this pronounced normalization of brain oscillations together with the significant decrease of the tinnitus frequency [Tass et al., 2012a] is indicative of plastic changes induced by the CR therapy.

### Comparison to Neurofeedback Training

Neurofeedback training is based on operant learning and aims at a mentally induced reinforcement of beneficial physiological processes mediated by a feedback of appropriate physiological variables [Sterman and Friar, 1972]. Initially, neurofeedback training in the treatment of tinnitus has been applied mainly with the aim to induce relaxation by upregulating posterior *α* [Gosepath et al., 2001; Schenk et al., 2005]. In that context, it has been suggested that distress generally is associated with a reduction of posterior *α* power and an enhancement of *β* power (14–30 Hz) [Gosepath et al., 2001; Schenk et al., 2005]. An upregulation of the *α* activity might, thus, be interpreted as a sign of increased relaxation. As a next step, regionally specific, presumably tinnitus‐related brain activity was used as feedback [Dohrmann et al., 2007a, 2007b]: Instead of posterior EEG sites, Dohrmann et al. (2007a, 2007b) used frontocentral EEG sites (F3, F4, FC1, and FC2, referenced to the right mastoid) as electrical generators located in the auditory cortex project to these frontal sites [Pantev et al., 1995].

Two different types of neurofeedback training with frontocentral EEG signals were used to attenuate tinnitus: *α*–*δ* training aims at a normalization of the spontaneous EEG by increasing *α* and decreasing *δ* power [Dohrmann et al., 2007a,2007b; Weisz et al., 2011]. In contrast, the goal of desynchronization suppression training is to maximize *α* power during sound stimulation and, hence, to mentally counteract the “natural” tendency of *α* desynchronization [Weisz et al., 2011]. However, both *α*–*δ* training [Dohrmann et al., 2007a,2007b; Weisz et al., 2011] and desynchronization suppression training [Weisz et al., 2011] lead to only a marginal modulation of *δ* power [Weisz et al., 2011]. Accordingly, it is still a matter of debate whether neurofeedback training specifically modulates tinnitus‐related brain activity, or rather simply affects nonspecific neuronal processes that influence tinnitus distress [Weisz et al., 2011]. Given our results of the comparison to baseline (i.e., prior to therapy; Tass et al., 2012a) as well as the comparison to healthy controls presented here, acoustic CR neuromodulation, in fact, specifically reverses the tinnitus characteristic EEG patterns by decreasing *δ* and *γ* and increasing *α* power in a tinnitus‐related network comprising auditory and nonauditory brain areas.

### Mechanism of CR Neuromodulation

In this study and in a previous study [Tass et al., 2012a], we analyzed the amount of neuronal synchronization (in terms of spectral power in EEG signals) before and after acoustic CR neuromodulation. In particular, we did not investigate the EEG dynamics during CR neuromodulation. Accordingly, as yet, we cannot determine in which way acoustic CR neuromodulation causes the long‐lasting desynchronization in widespread cortical neuronal populations. Based on the previous, in particular, computational studies, CR neuromodulation might cause a desynchronization of cortical neuronal populations in qualitatively different ways [Popovych and Tass, 2012; Tass and Popovych, 2012]. Given the tonotopic organization of the central auditory system [Ehret and Romand, 1997], and owing to the fact that phase resets may propagate via synapses [Jackson et al., 2002; Lerma and Garciaaustt, 1985; Perkel et al., 1964; Pinsker, 1977; Popovych and Tass, 2012; Prinz et al., 2003; Tass and Popovych, 2012], stimulation‐induced phase resets may, hence, occur at different hierarchical levels of the central auditory system. The tonotopic selectivity, that is, the spatial spread of the different stimuli crucially depends on the characteristics of the tuning curves of auditory nerve fibers, which may, for example, be pathologically broadened owing to a cochlear hearing loss [Givens, 1996; Perkel et al., 1964; Prinz et al., 2003; Ryan et al., 1979]. However, as shown computationally, CR neuromodulation is robust against variations of the spatial spread [Lysyansky et al., 2011]. For this reason, acoustic CR neuromodulation might, in principle, induce a desynchronization of the *δ* rhythm at the level of the primary auditory cortex by causing phase resets of the *δ* rhythm in different cortical subpopulations at different times. However, CR might cause a desynchronization at the cortical level also in a qualitatively different way. CR might cause a desynchronization of an upstream nucleus by its typical mechanism, that is, via time‐shifted phase resets of neuronal subpopulations [Tass, 2003], whereas further downstream in the central auditory system desynchronization might be achieved in a, so to speak, unspecific manner, that is, by a downstream propagation of the desynchronizing effect. Computationally it has been shown that desynchronizing effects may propagate from one neuronal population to another [Hauptmann et al., 2005; Popovych and Tass, 2010]. The “unspecific” propagation mechanism of desynchronization might be particularly relevant for the propagation of desynchronizing effects from auditory to nonauditory areas as this mechanism does not require a tonotopic organization of the connections. It is important to note that prior to CR therapy spatial peaks (i.e., local maxima) of the spectral power in the group of all bilateral tinnitus, patients were localized in the primary auditory cortex (BA 41) in the *δ* and low *γ* band (Fig. 3). In contrast, in the group of 12 good responders, the spatial peaks of the spectral power were found in the frontal lobe for *δ* (BA 44) and low *γ* (BA 46), whereas in the temporal lobes nearly all of the significantly different voxels vanished after 12 weeks of CR therapy (**RESULTS** and Fig. 3). This remarkable finding might be owing to the fact that the CR‐induced desynchronization first reaches the primary auditory cortex from where it propagates further downstream, into the tinnitus‐related network comprising different, and especially nonauditory cortical areas. A propagation process of this kind, which involves a larger network of neuronal populations, will likely take time, possibly in a similar way as it is known from, for example, propagation phenomena in oscillatory or excitable media [Mikhailov, 1990]. Accordingly, a complete evolution of this desynchronization propagation might, on average, require more than 12 weeks of CR therapy.

High levels of test retest reliability of quantitative EEG (e.g., LORETA) were demonstrated over many days and weeks [Cannon et al., 2012; Thatcher, 2010]. However, having performed only a single EEG recording in every subject from the control group might be considered as a limiting factor of this study as test–retest reliability of these recordings was not assessed. Accordingly, in future studies it may be desirable to assess the reliability of CR‐induced EEG normalization, observed in this study, using BESA source montage and sLORETA techniques to substantiate their role as an indicator of the course of tinnitus treatment.

## CONCLUSIONS

In this study, we focused on CR‐induced EEG power modulations and their association to the changes of the tinnitus sensation. CR‐induced modulation of the effective connectivity in a tinnitus‐related network, comprising auditory and nonauditory brain areas, was beyond the scope of this manuscript and will be investigated in a separate study [Silchenko et al., 2013]. In this study, we did not investigate the EEG dynamics during CR neuromodulation. Thus, as yet, we cannot determine the specific way that acoustic CR leads to a normalization of the oscillatory power in the tinnitus‐related network of brain areas. Further studies are necessary to further investigate the specific mechanisms of CR treatment and its comparison to other auditory stimulation paradigms. Accordingly, another study will be devoted to the dynamical mechanism of acoustic CR neuromodulation in tinnitus patients. To reveal the actual mechanism by which CR causes a desynchronization on the cortical level, we shall investigate in which auditory and nonauditory brain areas single tones cause a phase reset of pathological rhythms, for example, the *δ* rhythm, whereas the corresponding CR stimuli, that is, the time‐shifted sequences of phase resetting tones, cause a desynchronization. Also, further computational studies will be devoted to the propagation of desynchronizing effects in networks of interacting neuronal populations with different connection topologies to possibly optimize the stimulation protocol, for example, by identifying stimulation parameters that, properly chosen, might enable to substantially increase the propagation velocity. The goal of this combined theoretical and experimental approach is to optimize the CR therapy in a way that also the, fortunately, smaller portion of patients who still do not sufficiently profit from the current CR version might finally benefit as well.

## CONFLICT OF INTEREST

I. Adamchic and T. Toth have no conflict of interest in relation to this study. C. Hauptmann and P. A. Tass have a contractual relationship with ANM Adaptive Neuromodulation GmbH. P. A. Tass holds shares of ANM Adaptive Neuromodulation GmbH.

## Supporting information

Figure S1. Topographical maps of the group differences (tinnitus group vs tinnitus free control group) for power in the following frequency bands: 1–3.5 Hz (delta), 4–7.5 Hz (theta), 8–12 Hz (alpha), 12.5–30 Hz (beta), 30.5–48 Hz (low gamma) and 52–90 Hz (high gamma).
