# Supplementary figures and images for: Reversing pathologically increased EEG power by acoustic coordinated reset neuromodulation
Source: Hum Brain Mapp. 2013 Aug 1;35(5):2099–118. doi: 10.1002/hbm.22314 (PMC4216412; doi:10.1002/hbm.22314)

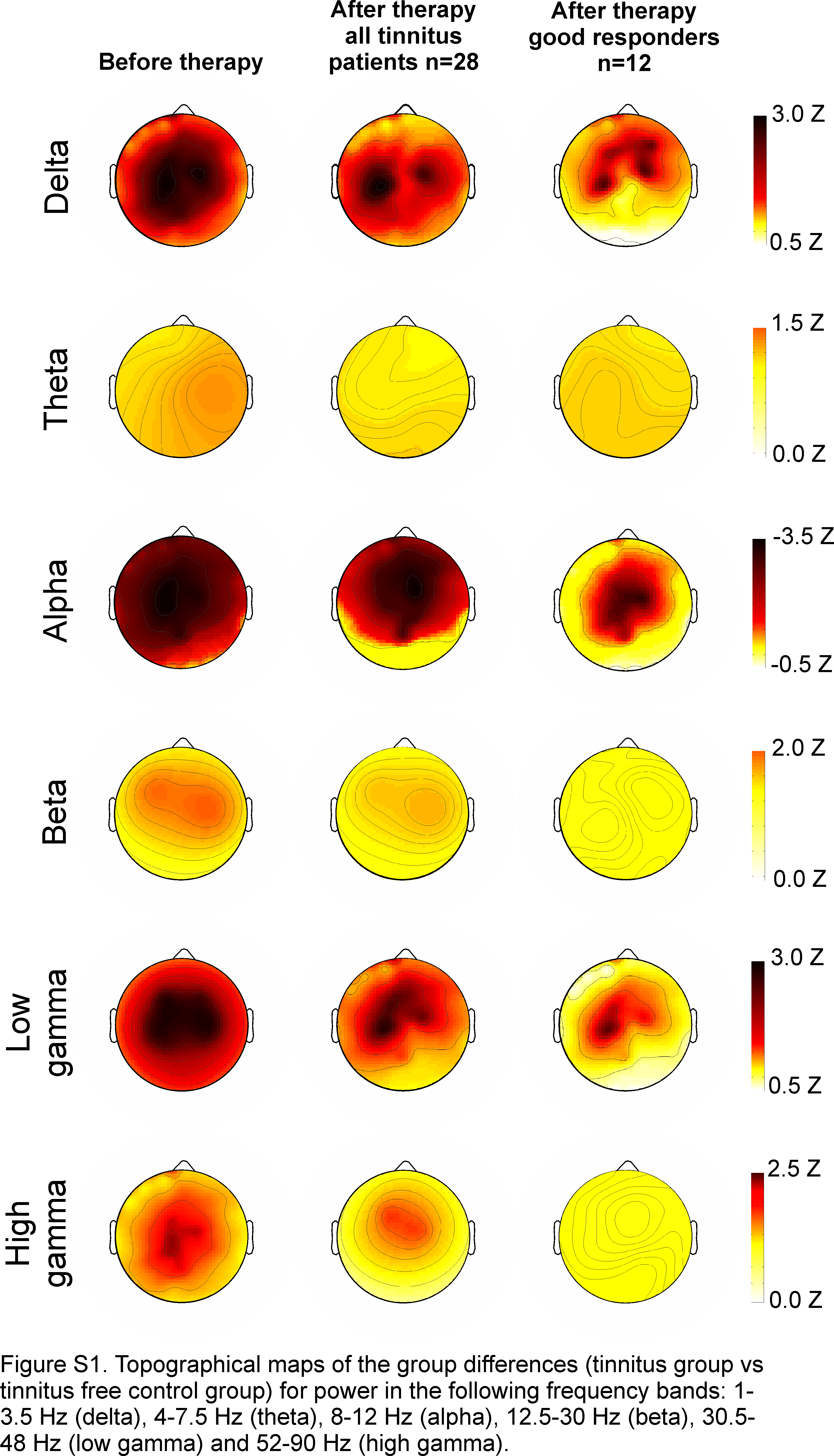

Supplement: Supplementary file 1 — Figure S1. Topographical maps of the group differences (tinnitus group vs tinnitus free control group) for power in the following frequency bands: 1–3.5 Hz (delta), 4–7.5 Hz (theta), 8–12 Hz (alpha), 12.5–30 Hz (beta), 30.5–48 Hz (low gamma) and 52–90 Hz (high gamma). [file HBM-35-2099-s001.tif]
